# The Effects of Exercise on Appetite-Regulating Hormone Concentrations over a 36-h Fast in Healthy Young Adults: A Randomized Crossover Study

**DOI:** 10.3390/nu15081911

**Published:** 2023-04-15

**Authors:** Landon S. Deru, Coleton J. Chamberlain, Garrett R. Lance, Elizabeth Z. Gipson, Benjamin T. Bikman, Lance E. Davidson, Larry A. Tucker, Jacob L. Coleman, Bruce W. Bailey

**Affiliations:** 1 Department of Exercise Science, Brigham Young University, Provo, UT 84602, USA; 2 Department of Cellular Biology and Physiology, Brigham Young University, Provo, UT 84602, USA

**Keywords:** fasting, exercise, appetite, incretins, ghrelin, GLP-1, hunger, satiety

## Abstract

Hunger and satiety are controlled by several physiological mechanisms, including pancreatic and gastrointestinal hormones. While the influence of exercise and fasting have been described individually, in relation to these hormones, there is a paucity of work showing the effects of the two modalities (fasting and exercise) combined. Twenty healthy adults (11 males, 9 females) completed both conditions of this study, each consisting of a 36-h water-only fast. One of the fasts began with treadmill exercise, and the differences between the conditions on various appetite hormones were measured every 12 h. The difference in the area under the curve between conditions for ghrelin was 211.8 ± 73.1 pg/mL (F = 8.40, *p* < 0.0105), and, for GLP-1, it was −1867.9 ± 850.4 pg/mL (F = 4.82, *p* < 0.0422). No significant differences were noted for areas under the curve between conditions for leptin, PP, PYY, insulin, or GIP. Initiating a fast with exercise lowers ghrelin concentrations and elevates GLP-1 concentrations. Given that ghrelin elicits feelings of hunger and GLP-1 signals feelings of satiety, adding exercise to the beginning of a fast may reduce some of the biological drive of hunger, which could make fasting more tolerable, leading to better adherence and more significant health outcomes.

## 1. Introduction

Dietary behaviors are linked to metabolic health and have been shown to predict non-communicable chronic diseases such as type 2 diabetes mellitus, heart disease, and cancer [1,2]. Research has linked adherence to a Western diet to the increased prevalence of insulin resistance, hyperinsulinemia, and obesity [3]. Additionally, high-frequency eating patterns observed in most westernized societies have led to a state of chronic inflammation and subsequent disease states [4], which can be combated through regular periods of abstinence from food [5].

Behaviors surrounding food consumption are influenced by a number of environmental, social, psychological, and physiological factors. This interplay between drivers of food consumption makes the control of dietary behaviors very complex and creates a challenge when attempting to regulate food intake. Physiologically, the drive to consume food is largely regulated by a balance of hormones that signal both satiety and hunger [6]. Some of these hormones include ghrelin, glucagon-like peptide-1 (GLP-1), peptide tyrosine-tyrosine (PYY), pancreatic polypeptide (PP), leptin, insulin, and glucose-dependent insulinotropic polypeptide (GIP). Ghrelin is a well-known gut hormone that stimulates feelings of hunger and the desire to consume food [7]. In contrast, gut hormones such as GLP-1, PYY, GIP, and leptin, as well as pancreatic hormones such as PP and insulin, are several among many appetite-reducing signals in the body [8]. These hormones are released postprandially from endothelial cells in the digestive tract and endothelial cells in the pancreas, respectively [9]. Increased concentrations of these hormones cause downstream cascades that reduce food seeking behaviors, limit food intake, and create a subjective feeling of satiety [10].

During a period of fasting, circulating levels of GLP-1, PYY, PP, GIP, leptin, and insulin are reduced, and levels of antagonistic hormones such as ghrelin and glucagon increase. These changes in hormones drive food-seeking behaviors [11]. In addition to their responses in states of feeding and fasting, each of these appetite-regulating hormones also fluctuates during and after exercise, with a general consensus that exercise temporarily induces appetite suppression [12,13]. This temporary decrease in appetite after exercise is correlated with increased concentrations of gut peptides PYY, GLP-1, GIP, and PP [14,15].

While previous works have assessed the independent effects of fasting and exercise on hunger, to date there are only a few that have evaluated the two in combination [16,17,18]. Hamilton et al. evaluated the impact of postexercise fasting on hunger and satiety using a randomized cross-over design in 14 adults. However, there was no non-exercise control group. The two experimental conditions included exercising at 65–75% of individual VO_2_ peak, and afterward participants either drank water or sweetened milk. The results of this study demonstrated that both GLP-1 and PYY decreased, and ghrelin increased, 1 h after the exercise bout in the water-only fasted condition, and that PYY, GLP-1, and ghrelin returned to baseline 12 h after the exercise bout in this condition [19]. The lack of a control group in this study is a major limitation, as there is no way to describe how the participants respond with fasting only.

We previously published the primary outcomes of this study. These results show that initiating a fast with a bout of exercise accelerated some of the metabolic changes that occur during fasting [20]. Specifically, exercise reduced the time to metabolic ketosis from 21.0 h to 17.5 h postprandially. However, performing an intense bout of exercise at the beginning of a fast to reduce time to metabolic ketosis may not provide a viable long-term strategy if it makes the fast more challenging or enhances the physiological drive to consume food. Thus, there is a need to describe the impact of combining fasting with exercise on the hormones that modulate the physiological drive to consume food. Specifically, this could inform individuals who participate in intermittent fasting and time-restricted eating protocols, since the length of the fast could be reduced by combining the two behaviors.

The aim of this study was to evaluate the impact of initiating a 36-h fast with or without a bout of exercise on plasma concentrations of ghrelin, GLP-1, GIP, PYY, PP, leptin, and insulin. We also describe the overall change in hormone concentrations over the course of a 36-h fast. These results represent secondary outcomes to data that has been previously reported [20].

## 2. Materials and Methods

The study was designed to compare the impact of fasting with and without an initiating bout of exercise on appetite hormones (PYY, PP, GLP-1, GIP, insulin, ghrelin, and leptin). To accomplish this purpose, we used a randomized crossover design with conditions counterbalanced. All participants were exposed to two treatment conditions. Both conditions took place on identical days of the week, and the washout between conditions was at least six days (the longest washout was 13 days). Both intervention days were identical, with the exception of the addition of an exercise bout that took place 30 min into the fast and after the standardized meal [21]. The study was approved by the university’s institutional review board (IRB2019-319).

Prior to beginning the study, randomizer.org was used to randomly assign the condition order to participant number [22] and participants were assigned numbers in chronological order as they joined the study. Before beginning any aspect of the study, participants were screened for any contraindications to participation and for eligibility based on inclusion criteria.

### 2.1. Participants

A total of 11 men and 9 women were recruited to participate in the study. Participants were recruited using advertisements, fliers, and word of mouth. Table 1 reports the demographic characteristics of the participants in the study. In order to take part in the study, participants had to be able to exercise at a vigorous intensity without restrictions. This was assessed using a physical activity readiness questionnaire (PAR-Q) [23].

Baseline physical activity was self-reported. Based on this self-report, 5% of participants participated in less than 30 min of exercise per week, 10% participated in 30 to 90 min of exercise per week, 35% participated in 90 to 150 min of exercise per week, 20% participated in 150 to 225 min of exercise per week, and 30% participated in more than 225 min of exercise per week.

The following are the exclusion criteria used for the study:

- Diagnosed with a metabolic disease;
- Diagnosed with an orthopedic impairment;
- Diagnosed with an eating disorder;
- Taking metabolism-altering medications [24];
- Consuming more than 60 mg of caffeine daily [25];
- Pregnant or lactating;
- Postmenopausal [26];
- underweight (BMI < 18.5 kg/m^2^) or obese (BMI > 30 kg/m^2^) [27];
- Practicing calorie or carbohydrate diets.

### 2.2. Measurements

#### 2.2.1. Anthropometric Measurements

Body mass and height were assessed at the beginning of each condition. Participants were assessed with their shoes removed and wearing a t-shirt and athletic shorts. Body mass was assessed using a digital scale accurate to ±0.1 kg (Seca, Hamburg, Germany). Height was assessed using a stadiometer (Seca, Hamburg, Germany) accurate to ±0.1 cm. Body composition was assessed on the initial visit using a GE iDXA (GE, Fairfield, CT, USA) [28]. The DXA was calibrated at the beginning of each assessment day per the manufacturer’s instructions. Encore version 17 was used to analyze the scans. 

#### 2.2.2. Plasma Hormone Levels

Blood draws were taken every 12 h of each fast (0, 12, 24, and 36 h). Samples were centrifuged for 15 min within 10 min of collection and then stored at −80 °F. Ghrelin, GLP-1, PYY, PP, leptin, insulin, and GIP were quantified using a standard 96-well multiplex human metabolic hormone magnetic bead panel according to the manufacturer’s instructions (EMD Millipore Corporation, Billerica, MA, USA, Catalog # HMHEMAG-34K).

### 2.3. Procedures

#### 2.3.1. Screening

Potential participants were emailed a link to an online survey, which was used to ensure all inclusion criteria for the study were met. In the survey, participants were asked to complete the PAR-Q, a food preference questionnaire, and report any food allergies. Candidates who qualified were invited to participate in the study. They were instructed to arrive at the lab prepared to exercise, and to refrain from vigorous activity for 24 h prior to testing, along with avoiding caffeine and other stimulant consumption. Adherence to these protocols was assessed through verbal questioning at the beginning of each testing session. Adherence was later reaffirmed through the assessment of baseline insulin, which was found not to differ between conditions, suggesting that participants began each condition of the study in a similar metabolic state. If participants were non-compliant with pretest protocols, they were rescheduled.

#### 2.3.2. Orientation

Participants gave informed consent prior to participation in the study. Each participant was familiarized with the study procedures and informed of the purpose of the study. During the testing period, participants were asked to go about their normal daily activities while avoiding strenuous activity, including cardiovascular or strength training, hiking, yard work, or other forms of vigorous exercise or activity. Participants were also asked to maintain their regular sleeping patterns during the testing period. Dietary and sleep adherence were verified using an activity and sleep log and were also verbally verified at each visit.

#### 2.3.3. Standardized Meals

To initiate each fast, a standardized meal was given to each participant. Equations validated for reliability and accuracy by Hall et al. [29] were used to estimate the energy needs of each participant. The equation predicts basal metabolic rates (BMR) using weight (kg), height (cm), age (years), and sex. To estimate daily total energy requirements, an activity factor of 1.55 was used [30]. This activity factor represents an estimate of daily energy expenditure during physical activity throughout both work and leisure time. An activity factor of 1.55 represents someone who is largely sedentary at work and does some moderate activity outside of work 1–2 times per week [27]. The meals had a standardized macronutrient content (60% carbohydrate, 15% protein, and 25% fat) and contained combinations of beef jerky, apple slices, crackers, string cheese, raw almonds, and pre-packaged peanut butter and jelly sandwiches. Meals were assembled in the research lab using commercially available boxed and prepackaged foods that were weighed to match the energy and macronutrient composition requirements of each participant. The standardized meal provided 25% (BMR × 1.55 × 0.25) of the participants’ daily caloric requirements. The meal was the same for each participant on both fast initiation days, and participants were asked to consume all the food in the meal. Adherence to meal consumption was assessed by direct observation in the research lab. Participants were scheduled in a manner that allowed the standardized meal to be consumed at the same time in both conditions. On the non-exercise day, the participants arrived at the lab approximately 20 min earlier than on the exercise day to allow sufficient time for a DXA scan to take place prior to the blood draw and standardized meal.

#### 2.3.4. Treatment Sessions

Participants were asked to maintain normal eating habits leading up to each fast and to abstain from eating for at least four hours before consuming the standardized meal at the initiation of their fast in an effort to normalize blood markers and prevent pre-fast caloric loading [31]. Demographic information, anthropometric data, and consent were all obtained before initiating the intervention, and researchers reviewed testing protocols and procedures with each participant before initiating each fasting session. After these screenings and anthropometric measurements, a venous blood draw was performed, followed by the consumption of a standardized meal to begin the 36-h fast. Blood draws and anthropometric measurements were taken every 12 h during the fast (12, 24, and 36 h), and participants were reminded to stay hydrated throughout the duration of the fast. These were water-only fasts, meaning that no other caloric or non-caloric food or beverages were allowed while fasting, including drink additives and gum chewing.

#### 2.3.5. Exercise Protocol

Exercise protocols were completed on a treadmill at a speed and grade designed to bring the estimated heart rate reserve (HRR) of each participant to 70%. This type of exercise is considered intense [32] and was designed to maximize glucose oxidation compared to exercise at lower intensities [33]. The formula used to calculate the target heart rate for each participant was [34]:

Target HR = [% target intensity × (HR_max_ − HR_rest_)] + HR_rest_ (1)

The following formula was used to estimate maximal HR [35]:

HR_max_ = 208 − (0.7·age) (2)

A strap-on heart rate monitor (Garmin, Olathe, KS, USA) was fitted to the participant, and they were then instructed to sit for 5 min to establish their resting HR (HRrest). Once HR calculations were complete, subjects began the exercise. On the treadmill, the speed and grade were adjusted and set to cause the participant to meet their target HR within 5 min after beginning exercise. Once set, the speed and grade were not adjusted. Participants were expected to maintain this intensity for the entire duration of the exercise; however, if a participant was unable to maintain the exercise intensity, they were permitted a 60-s break before resuming.

The exercise duration was individualized in order for each participant to expend approximately the same number of calories as they consumed in the standardized meal. The energy expenditure calculation was based on the ACSM metabolic equation, which converts oxygen to calories by multiplying liters of oxygen by 5.

Standard ACSM metabolic equation:

VO_2_ mL·kg^−1^ min^−1^ = (0.2·S) + (0.9·S·G) + 3.5 (3)

Equations to estimate exercise duration:

min = (E/5·1000)/(kg((0.2·S) + (0.9·S·G) + 3.5))(4)

E = energy from a meal (kilocalories);

kg = participant’s weight (kilograms);

S = treadmill speed (meters per minute);

G = grade of the treadmill (percent incline);

min = time on the treadmill (minutes).

All calculations were performed in a protected spreadsheet using the above preset equations to ensure accuracy. Energy expenditure was verified using indirect calorimetry throughout the entire duration of the exercise (COSMED, Rome, Italy).

### 2.4. Statistical Analysis

Means and standard deviations were used to describe participant data. The primary factors in the statistical models were condition and sex. The two levels of the condition variable were fasting alone and fasting with exercise. The sample size was calculated a priori. Using an alpha of 0.05, a beta of 0.20, and an effect size of 0.66 (a ten percent difference between conditions) resulted in a sample of 20 participants.

A mixed-model repeated-measures ANOVA was used to evaluate the time course differences for each hormone over the 36-h fast for each condition, controlling for baseline values. Where there were significant interactions between time and condition, a post hoc least significant difference pairwise comparison was used to examine the differences. Gender was included in the model as a covariate, and the interactive affect between gender and condition was evaluated. The area under the treatment curve was calculated using the trapezoidal rule for the plasma concentration of each hormone. The area under the curve was used to better represent the total response of each variable. A mixed-model repeated-measures ANOVA was used to compare areas under the curve for each condition. The cut point for statistical significance was *p* ≤ 0.05. SAS software version 9.4 for Windows was used to analyze the data.

## 3. Results

**Figure 1:**
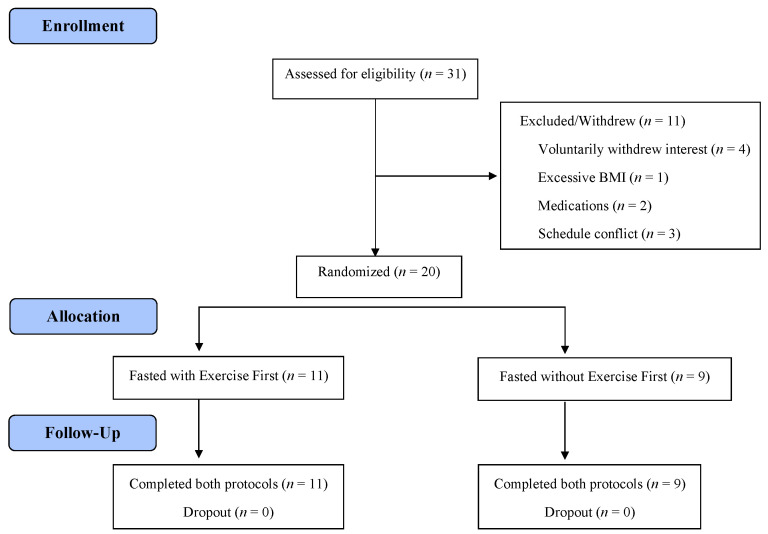
Participant flow diagram.

Thirty one applicants were screened for qualification (Figure 1). Eleven men and nine women were randomly assigned to a condition order, and each completed all aspects of the study. Figure 1 describes the demographics of the participants. The standardized meal fed to participants prior to each fasting session consisted of 614.8 ± 85.2 kcal. The measured energy expenditure during exercise was 587.6 ± 120.1 kcal, and the average metabolic equivalents (MET) of the exercise bout were 9.14 ± 1.37. One MET describes the amount of oxygen consumption by the body at rest, and multiples of this value help quantify relative energy expenditure [36]. The average respiratory quotient (R) during the exercise bout was 0.95 ± 0.14, indicating that glucose acted as the primary metabolic fuel [33].

**Sex differences.** Eleven men and nine women participated in the study to completion. Men tended to have higher plasma insulin concentrations than women (F = 9.66 and *p* = 0.0064). There was no observed difference between men and women for any of the other biomarkers. Additionally, there was no significant difference between men and women in their responses to the intervention. Specifically, there was no sex-by-condition interaction observed for ghrelin (F = 0.02, *p* = 0.8848), GLP-1 (F = 1.93, *p* = 0.1837), leptin (F = 1.38, *p* = 0.2570), PP (F = 0.98, *p* = 0.3381), PYY (F = 0.09, *p* = 0.7684), insulin (F = 0.01, *p* = 0.9069), or GIP (F = 0.01, *p* = 0.9379). Because men and women responded similarly to the intervention, they were analyzed together.

**Differences between conditions.** There was no significant difference between conditions at the beginning of each fast for any of the hormones. There was a significant condition by time interaction for ghrelin (F = 3.22, *p* = 0.0457; see Table 2). All other condition by time interactions were not significant. In addition, after the initiation of the fast, there was a main effect on condition for ghrelin (F = 4.14, *p* = 0492) and GLP-1 (F = 4.78, *p* = 0.0352; see Table 2). Follow-up analysis shows that ghrelin was significantly higher when fasting alone vs. fasting with exercise at hours 12 (t = 2.51, *p* = 0.0141) and 24 (t = 2.26, *p* = 0.0264). In contrast, GLP-1 was significantly lower in the fasting and exercise condition at hour 36 (t = −1.99, *p* = 0.0499).

**Figure 2:**
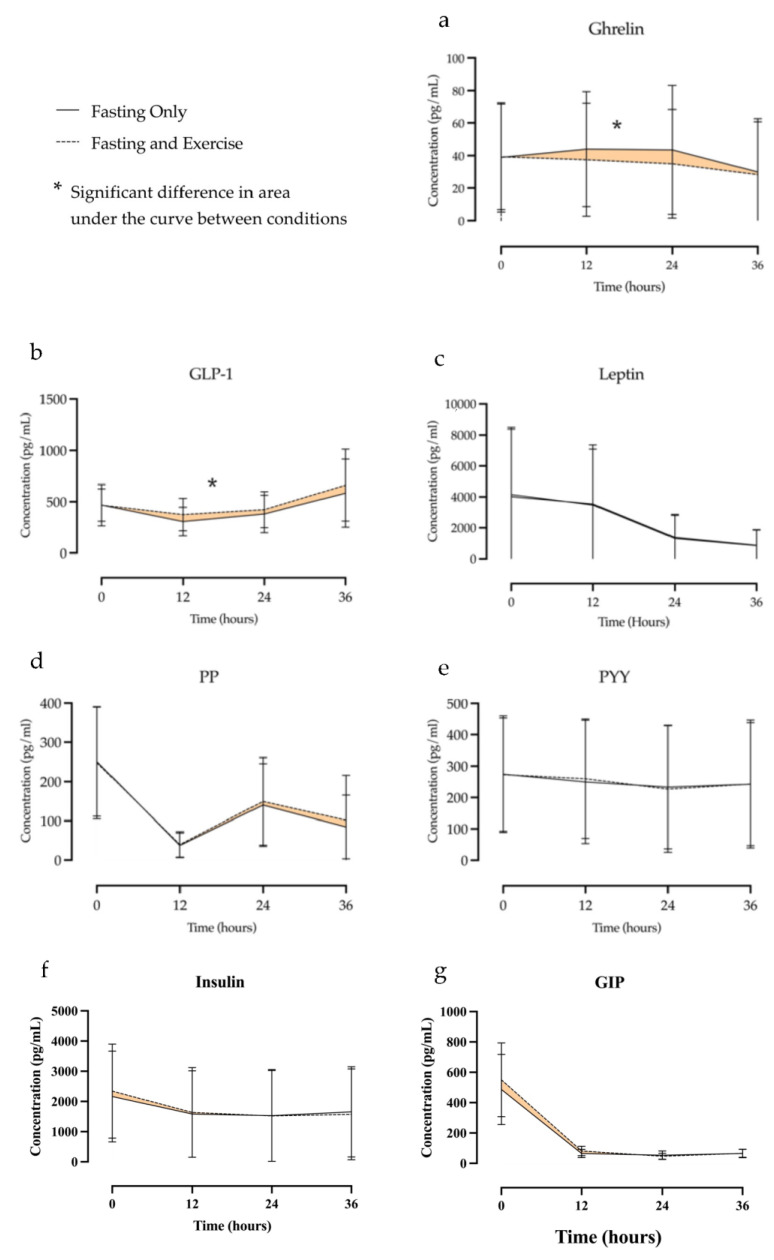
Concentrations of ghrelin (**a**), GLP-1 (**b**), leptin (**c**), PP (**d**), PYY (**e**), insulin (**f**), and GIP (**g**) over the course of a 36 h fast. Differences in area under the curve are highlighted.

**Area under the curve between conditions.** Figure 2 presents the hormonal response to each condition and highlights the area difference between conditions. Table 3 presents the differences in area under the curve between conditions for each of the measured hormones. Significant differences between conditions were noted for both ghrelin and GLP-1. The difference in area under the curve for ghrelin was 211.8 ± 73.1 pg/mL (F = 8.40, *p* < 0.0105) with ghrelin levels being higher during fasting only condition (Figure 2A). This represented a 17% difference in ghrelin concentration between conditions over the course of the fast. The difference in area under the curve for GLP-1 was −1867.9 ± 850.4 pg/mL (F = 4.82, *p* < 0.0422) with GLP-1 levels being lower during the fasting-only condition (Figure 2B). This represented a 13% difference between conditions. No significant differences were noted for areas under the curve between conditions for leptin, PP, PYY, insulin, or GIP (Figure 2c–g).

**Changes in appetite biomarker levels over time due to fasting. **Table 2 presents the changes in concentration over time for each of the measured hormones in each condition. Plasma ghrelin concentrations remained constant for the first 24 h of the fast and then decreased from 24 to 36 h (t = 4.01, *p* < 0.0001). Plasma GLP-1 concentrations were lower than baseline at hours 12 (t = 3.84, *p* < 0.0002) and 24 (t = 2.02, *p* = 0.046), then rebounded above baseline levels at 36 h (t = −4.61, *p* < 0.0001). In contrast, plasma leptin concentrations were progressively lower at each time point over the 36-h fast (F = 38.37, *p* < 0.0001). Plasma PP concentrations were lower than baseline at 12 (t = 11.77, *p* < 0.0001), 24 (t = 5.56, *p* < 0.0001) and 36 h (t = 8.70, *p* < 0.0001). However, at 24 (t = −6.25, *p* < 0.0001) and 36 h (t = −2.97, *p* < 0.0036), PP concentrations were higher compared to hour 12 but remained lower than baseline. Concentrations of PYY decreased from baseline at 12 (t = 2.59, *p* = 0.0111) and 24 h (t = 5.56, *p* < 0.0001) and then did not change from 24 to 36 h (t = −1.54, *p* = 0.1264). Insulin concentrations decreased from baseline over the first 12 h (t = 8.54, *p* < 0.0001) and then did not change between 12 and 24 h (t = 0.84, *p* = 0.4040) or between 24 and 36 h (t = −0.63, *p* = 0.5300). Finally, concentrations of GIP decreased at 12 h from baseline (t = 11.57, *p* < 0.0001) and then did not change between 12 and 24 h (t = 0.32, *p* = 0.7511) or between 24 and 36 h (t = −0.33, *p* = 0.7432).

## 4. Discussion

Recent research indicates that fasting may lead to weight loss but may also increase concentrations of ghrelin while reducing concentrations of GLP-1, PYY, and PP throughout the fast [37]. These hormonal changes can increase the drive to consume food, making it difficult to participate in fasting. The purpose of this study was to determine how exercising at the beginning of a 36-h fast alters hormones related to hunger and satiety. We were interested in evaluating the combination of exercise and fasting since exercise has the potential to accelerate the depletion of liver glycogen, causing the body to upregulate fat utilization and ketone production. While this switch in substrate utilization can have a positive impact on health, the addition of exercise to fasting may alter feelings of hunger and the drive to consume food.

We found that completing a bout of vigorous exercise at the beginning of a fast depressed concentrations of ghrelin by 17% while raising concentrations of GLP-1 by 13%, an effect independent of biological sex. In contrast, PYY, PP, leptin, and insulin were not altered with exercise. These results help describe the impact of acute exercise on appetite and generally agree with the notion that exercise may be a mild to moderate anorexigenic stimulus [12].

Previous studies have examined how different exercise intensities and durations can affect GLP-1 by examining post-exercise GLP-1 concentrations. For example, Martins et al. reported that total plasma GLP-1 concentration increased between 16–70% an hour after exercising at 60% of max heart rate [14]. Similarly, Holliday and Blannin found that GLP-1 concentrations were elevated by 17% after high-intensity interval training in 8 participants 90 min post exercise, though these findings did not reach significance, likely due to the limited sample size [38]. Finally, Ueda et al. observed a 17% rise in GLP-1 concentration 30 min post exercise at both moderate (50% VO2 max) and high intensity (75% VO2 max) [39]. The mechanism driving this change in GLP-1 is not currently well described. However, Hamasaki suggests that a potential mechanism for the improved GLP-1 secretion with exercise may be due to the increased production of microbiota-derived short chain fatty acids [40]. These fatty acids interact with G-protein-coupled receptors on the intestinal L-cells, which stimulates an increase in GLP-1 production [41].

Because blood was taken every 12 h, our study was not designed to capture the acute effects of exercise. In contrast to the acute post-exercise studies described previously, the current study demonstrated that GLP-1 was suppressed in both conditions 12 to 24 h into the fast. However, the findings from this study agree with previous research in that exercise elevated GLP-1 by comparison to the control condition. One of the unique contributions of this study is that we observed the elevation of GLP-1 beyond the 2 h post-exercise seen in other research, demonstrating that the impact of exercise on GLP-1 lasted the duration of the fast. Interestingly, between 24 and 36 h of fasting, GLP-1 rose above baseline in both conditions, suggesting that the impact of fasting on GLP-1 is not linear, though the mechanism describing the rise in GLP-1 after 24 h of fasting remains uncertain [42].

Similar to GLP-1, multiple studies have evaluated how moderate to intense exercise influences ghrelin. However, the findings from these studies are less consistent [43]. Holliday et al. observed a short-lived suppression of ghrelin after an acute bout of moderate to vigorous exercise [38]. Similarly, Broom et al. found that exercise at a vigorous intensity decreased ghrelin [44]. In contrast, Erdmann et al. found that ghrelin concentrations did not change with high intensity exercise [45], and Burns et al. found no difference in ghrelin concentrations up to 1 h after moderate-high intensity running despite subjective hunger being suppressed [46].

We demonstrated that exercise mitigates the rise in ghrelin typically observed with fasting, as there was no change in baseline ghrelin concentrations for the first 24 h of the fast in the exercise condition. Our study agrees with most of the current research because, relative to the control, adding exercise to the fast reduced ghrelin concentrations. Though there are significant differences between the experimental conditions during the first 24 h of the fast, our results indicate that the impact of exercise on ghrelin during a fast disappears by 36 h.

While additional work is needed to describe the mechanisms responsible for changes in ghrelin in response to acute exercise [43], some potential mechanisms have been proposed. One of the speculated mechanisms for this change in ghrelin is the post-exercise buildup of lactate, which is thought to suppress ghrelin by inhibiting ghrelin-mediated calcium mobilization in cells expressing growth hormone secretogogue receptors [47]. These G-protein coupled receptors are found throughout the body and play a key role in determining energy balance, food intake, and metabolism [48]. Thus, inhibiting these receptors may act to mitigate hunger [47]. Another potential mechanism for the observed decrease in ghrelin after exercise may be due to the sympathetic response of exercise, which decreases activation of the vagus nerve and slows gastric motility and the release of gastric peptides, including ghrelin [49]. However, these mechanisms only describe the acute effects of exercise on ghrelin. Further work is needed to explain the observations of this study, where ghrelin was lower in the exercise condition between 12 and 24 h of fasting.

Several studies have also shown that aerobic exercise can alter plasma concentrations of PYY [50], PP [42], GIP [51], and leptin [52], though the impact of exercise on these hormones is transient. For example, PYY concentrations appear to be lower for only 90 min following exercise [53]. In contrast, PP has been shown to be elevated in a number of studies during and immediately following acute exercise, but not beyond this time frame [42]. GIP concentrations seem to peak approximately 30 min after exercise and return to near baseline by 180 min after exercise [51]. Leptin levels tend to return to baseline an hour following exercise [52]. Our results support these findings and indicate that by 12 h any potential hormonal shifts from exercise that may have occurred in these hormones are gone.

The results from this study show that exercise also did not alter insulin concentrations following 12 h of fasting. Physical activity is considered a means of improving insulin sensitivity, which stems from increased membrane permeability and glucose transport at the skeletal muscle independent of insulin [54,55]. Additionally, after bouts of moderate- and high-intensity exercise, plasma concentrations of insulin typically increase [56]. Elevated concentrations of insulin after exercise support the notion that exercise can decrease sensations of hunger and food-seeking behaviors [57]. Since our study did not measure the acute effects of exercise, we did not observe any elevations in insulin; rather, we saw insulin drop by 12 h and remain low for the remainder of the fast. Any increases in insulin that may have occurred post-exercise were likely transient.

A host of evidence suggests that hunger increases over the course of a fast. This is based on subjective ratings of hunger as well as alterations in hormones such as ghrelin, GLP-1, PYY, PP, GIP, and leptin [58,59]. We observed that PYY, PP, GIP, leptin, and insulin were all reduced compared to baseline for the full 36-h fast, while ghrelin was elevated, and GLP-1 was suppressed only during the first 24 h. These changes in satiety-related hormones are aligned with the mechanisms in the body to increase energy intake and restore energy balance when food is limited.

After 24 h, GLP-1 rebounded to concentrations higher than baseline, and ghrelin concentrations dropped below baseline. The exact impact of these two hormones on hunger and the drive to consume food is complicated since other appetite-suppressing hormone concentrations remained low throughout the duration of the fast. However, the changes in GLP-1 and ghrelin may partially mitigate the drive to consume food and could at least partially explain why hunger is highest during the first 24 h of a fast [60].

Results from our lab recently found that a state of nutritional ketosis could be reached approximately 3.5 h sooner when a fast was initiated with exercise [20]. The metabolic substrate switching associated with fasting can be achieved more quickly with exercise at the beginning of a fast without any negative impact on hormones that drive hunger. In fact, these results suggest that exercise may partially reduce the biological drive to consume food during a fast. These outcomes can inform best practices for individuals observing intermittent fasting and time-restricted eating patterns. Our findings suggest that individuals who exercise at the beginning of their fast may accelerate the metabolic benefits with minimal impact on concentrations of physiological drivers of hunger as well as subjective feelings of hunger. Thus, some of the metabolic benefits of fasting could feasibly be achieved in a shorter timeframe or to a greater extent using this strategy.

### Limitations and Strengths

This was an acute study evaluating the change in appetite hormones over the course of a single 36-h fast. While the results are informative, we are unable to describe how these results may change with repeated fasts over time. In addition, while each participant abstained from food for four hours prior to presenting at the lab and was fed a standardized meal at the beginning of each fast, the food intake prior to that was not controlled. The food and drink consumed prior to that point may have had a small residual impact on the participant’s metabolic state. However, participants were instructed to follow normal eating patterns and not overconsume at these meals in anticipation of the fast. There were no differences between appetite hormones at baseline, indicating that participants started each fast in a similar metabolic state. The fast was performed under free-living conditions, which lack the control of a lab environment. However, while this introduces some variability between fasts, the results are more generalizable since behaviors were not constrained. In addition, condition order was randomized, and any random differences in behavior between conditions should be controlled. Finally, blood draws were taken every 12 h, occurring at baseline, 12, 24, and 36 h. More frequent blood samples would better describe the time course of metabolic changes during the fast, especially within the first few hours after exercise. Additionally, we did not control the phases of the menstrual cycle of the women in the study. This could allow for more variability in appetite hormones since the hormone concentrations change with the phase of the menstrual cycle [61]. However, the pre-fast hormone concentrations were not different between conditions, and the response to the intervention was not different between men and women. Finally, we did not have a non-fasted and non-exercised normal living control over the course of the 36-h period. This limits the conclusions that can be drawn from the study about time-course changes in hormone concentrations since we were not able to contrast our findings to what would occur in a non-fasted state.

Despite these limitations, this study adds to the current literature regarding the effects of combined fasting and exercise. While most research has evaluated the impact of fasting or exercise on appetite-regulating hormones separately, this study is one of the first to evaluate the behaviors in combination. In addition, most studies only evaluate a few hormones at a time or each hormone individually. The current study described the impact of exercise and fasting on seven hormones, which allows a broader perspective on how fasting and exercise influence the biological drive to consume food. While much of the existing literature captures the acute impact (30 min to 3 h) of exercise or fasting on appetite hormones, this study evaluated the impact up to 36 h. This enabled us to describe the prolonged effects of exercise on these hormones and to observe the non-linear response of both GLP-1 and ghrelin. Finally, the practice of intermittent fasting and time-restricted eating is very popular because of their potential to improve body composition and metabolic health. This study adds to the current body of literature by describing how exercise could be used in conjunction with fasting to potentially enhance the benefits of this behavior while mitigating the negative effects of hunger.

## 5. Conclusions

This study found that initiating a fast with a bout of vigorous treadmill exercise lowers ghrelin and elevates GLP-1 concentrations compared to fasting alone. However, the relationship between hormone concentration and hunger seems to be complicated and the changes that we observed in GLP-1 and ghrelin did not seem to be enough to alter the subjective rating of hunger. These results, coupled with our previously reported findings [20], indicate that starting a fast with exercise accelerates the metabolic substrate switching observed during a fast without negatively impacting the subjective and physiological drives to consume food. These results may educate individuals participating in a variety of approaches to fasting (i.e., intermittent fasting, alternate day fasting, or time-restricted feeding), and potentially improve adherence and sustainability over time. Future research should evaluate these findings longitudinally to describe if there are any adapted responses associated with sustained participation in these behaviors. In addition, future studies should evaluate how different exercise modalities (strength training, high-intensity interval training, calisthenics, etc.) impact these hormones.

## Figures and Tables

**Table 1:** Demographic characteristics of participants.

	Male (*n* = 11)		Female (*n* = 9)		Cumulative (*n* = 20)	
	Mean	SD	Mean	SD	Mean	SD
Age (years)	26.5	7.1	25.8	4.3	26.2	5.8
BMI (kg/m^2^)	24.7	3.1	22.7	3.6	23.8	3.4
BF %	18.8	6.3	27.8	4.5	22.9	7.1
Visceral Adipose (g)	443.6	275.5	133.5	116.6	304.0	265.9
Exercise Time (min)	49.3	5.9	54.7	7.8	51.7	7.2
Ethnicity	*n*	**%**	*n*	**%**	*n*	**%**
Asian	1	9	2	22	3	15
Caucasian	8	73	7	78	15	75
Hawaiian/Pacific Islander	2	18	0	0	2	10

**Table 2:** Hormone concentrations (pg/mL) at four time points during a 36 h fast with or without exercise.

Hormone	Condition	0 h *	12 h	24 h	36 h	F-Value	*p*-Value
Insulin (pg/mL)	No Exercise	2163.7 ± 1501.9 ^a^	1584.3 ± 1429.9 ^b^	1530.4 ± 1529.1 ^b^	1654.7 ± 1495.4 ^b^	0.79	0.4568
	Exercise	2343.9 ± 1556.8 ^a^	1635.8 ± 1484.8 ^b^	1522.8 ± 1506.6 ^b^	1572.6 ± 1506.0 ^b^		
GIP (pg/mL)	No Exercise	486.7 ± 231.8 ^a^	65.0 ± 27.6 ^b^	53.4 ± 28.6 ^b^	65.4 ±28.4 ^b^	2.82	0.0658
	Exercise	550.7 ±242.4 ^a^	81.8 ± 30.9 ^b^	47.3 ± 19.9 ^c^	66.0 ± 26.5 ^d^		
GLP1 (pg/mL)	No Exercise	454.1 ± 126.9 ^a^	307.5 ± 139.3 ^b^	381.1 ± 183.6 ^b^	563.4 ± 287.1 ^c†^	0.20	0.8226
	Exercise	450.8 ± 143.4 ^a^	387.1 ± 158.8 ^b^	447.5 ± 185.2	672.9 ± 344.8 ^c^		
Ghrelin (pg/mL)	No Exercise	38.9 ± 31.9 ^a^	45.9 ± 35.4 ^b†^	44.1 ± 38.5 ^b†^	28.4 ± 31.3 ^c^	3.22	0.0457
	Exercise	38.0 ± 32.2 ^a^	31.8 ± 25.0 ^ab^	31.4 ± 26.8 ^ab^	28.2 ± 31.5 ^b^		
PP (pg/mL)	No Exercise	251.0 ± 138.9 ^a^	37.7 ± 31.5 ^b^	140.4 ± 105.3 ^c^	84.4 ± 81.3 ^b^	0.09	0.9107
	Exercise	248.1 ± 142.8 ^a^	40.2 ± 32.8 ^b^	149.7 ± 111.5 ^cd^	101.9 ± 114.4 ^d^		
PYY (pg/mL)	No Exercise	274.2 ± 186.1 ^a^	249.6 ± 196.5 ^b^	233.5 ± 196.9 ^c^	242.6 ± 195.8 ^bc^	1.79	0.1750
	Exercise	272.9 ± 180.7 ^a^	259.7 ± 190.1 ^b^	227.0 ± 201.7 ^c^	242.9 ± 203.5 ^d^		
Leptin (pg/mL)	No Exercise	4015.2 ± 4488.0 ^a^	3542.3 ± 3820.6 ^b^	1382.0 ± 1426.8 ^c^	868.9 ± 1007.6 ^d^	0.00	0.9954
	Exercise	4152.3 ± 4235.3 ^a^	3481.2 ± 3609.3 ^b^	1307.0 ± 1562.8 ^c^	844.8 ± 1011.3 ^d^		

Mean ± standard deviation. F and *p*-values are referring to the condition by time interaction for each outcome. * No difference between conditions at baseline. ^a–d^ Refer to the follow-up analysis for the main effect of time. Different letters in the same row indicate significantly different means (*p* < 0.05). ^†^ Indicates a significant difference between conditions at the given time point for ghrelin and GLP-1 (*p* < 0.05).

**Table 3:** Differences in area under the curve for fasting with and without exercise over 36 h.

	Fasting and Exercise (pg/mL^2^)	Fasting without Exercise (pg/mL^2^)	Change Scores	F-Values	*p*-Values
Ghrelin	1251.2±1170.7	1455.8±1190.8	−204.6	8.40	0.0105
GIP	5249±1938.7	4713.1±1717.7	535.9	0.34	0.5676
GLP1	16360±6474.6	14,399.8±5738.9	1868	4.82	0.0422
Insulin	61,403.1±53,781.1	59,790.5±53,095.8	1612.6	2.90	0.1070
PP	4378.6±2492.7	4075.6±2232.5	303	2.92	0.1055
PYY	8935.1±6979.5	8898.2±6990.8	36.9	1.22	0.2879
Leptin	89,348 ± 3471.94	86,018 ± 3473.66	3330.80	0.66	0.4287

## Data Availability

The data supporting the reported results are available on the Open Science Framework at https://osf.io/vm7ba/ accessed on 13 March 2023.
